# Supplementary material for: Bipolar Membrane Electrodialysis for Direct Conversion of L-Ornithine Monohydrochloride to L-Ornithine
Source: Int J Mol Sci. 2023 Aug 24;24(17):13174. doi: 10.3390/ijms241713174 (PMC10488261; doi:10.3390/ijms241713174)
Supplement: Supplementary file 1 [file ijms-24-13174-s001.zip › ijms-2552923-supplementary.pdf]

International Journal of
Molecular Sciences

Supplementary Materials

Bipolar Membrane Electrodialysis for Direct Conversion of L-Ornithine Monohydrochloride to L-Ornithine

Jinfeng He [1,2,†], Wenlong Liu [1,†], Jianrong Hao [1], Xixi Ma [1], Zhiyi Zheng [1], Yinghan Fang [1], Yuxin Liang [1], Zhihao Tian [1], Li Sun [1,2], Chuanrun Li [1,2,*] and Haiyang Yan [1,2,*]

[1] Pharmaceutical Engineering Technology Research Center, School of Pharmacy, Anhui University of Chinese Medicine, Hefei 230012, China
[2] Anhui Province Key Laboratory of Pharmaceutical Preparation Technology and Application, Hefei 230012, China
* Correspondence: crli@ahtcm.edu.cn (C.L.); oceanyan@ustc.edu.cn (H.Y.)

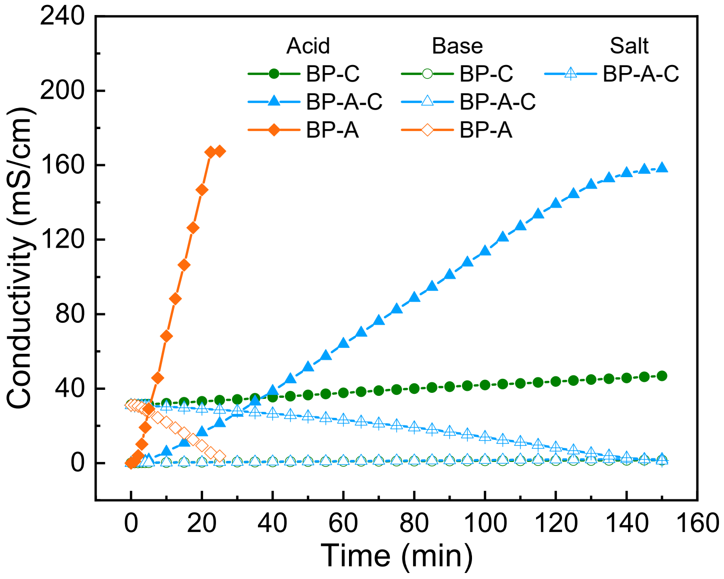

Figure S1. Conductivities of acid, base and salt solutions as a function of time for various stack configurations.

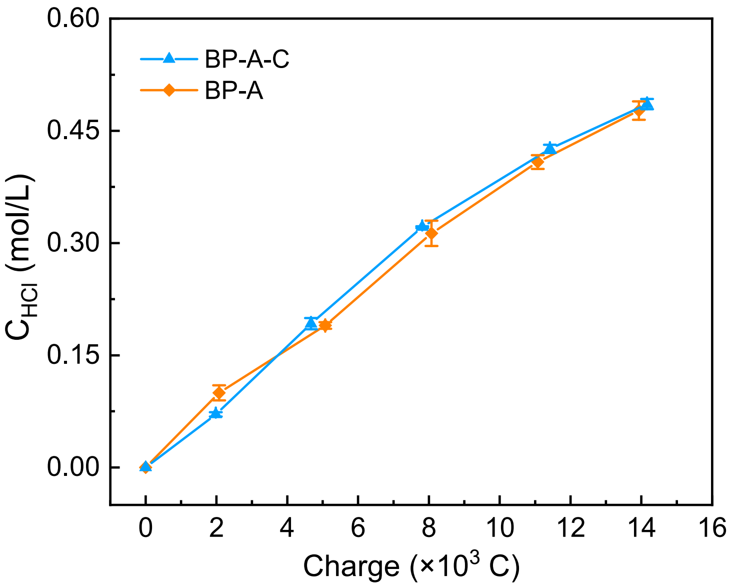

Figure S2. The concentration of HCl in the acid compartment (C_{HCl}) as a function of charge for various configurations.

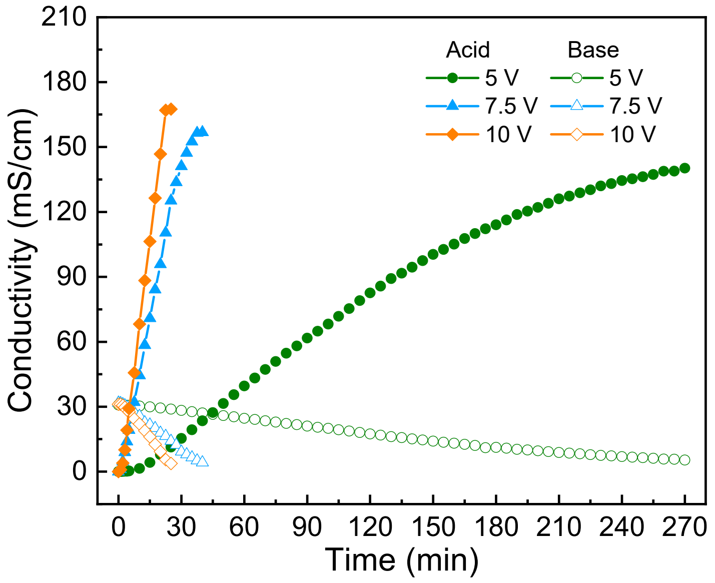

Figure S3. Conductivities of acid and base solutions as a function of time for various voltage drops.

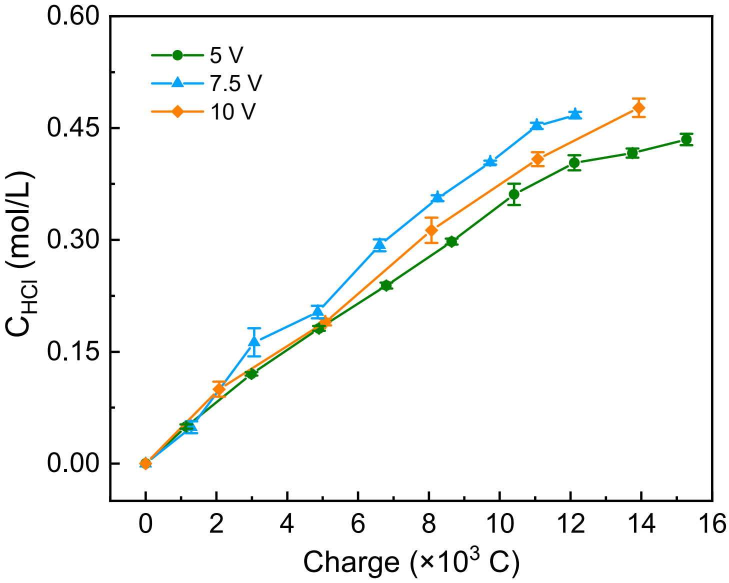

Figure S4. The concentration of HCl in the acid compartment (C_{HCl}) as a function of charge for various voltage drops.

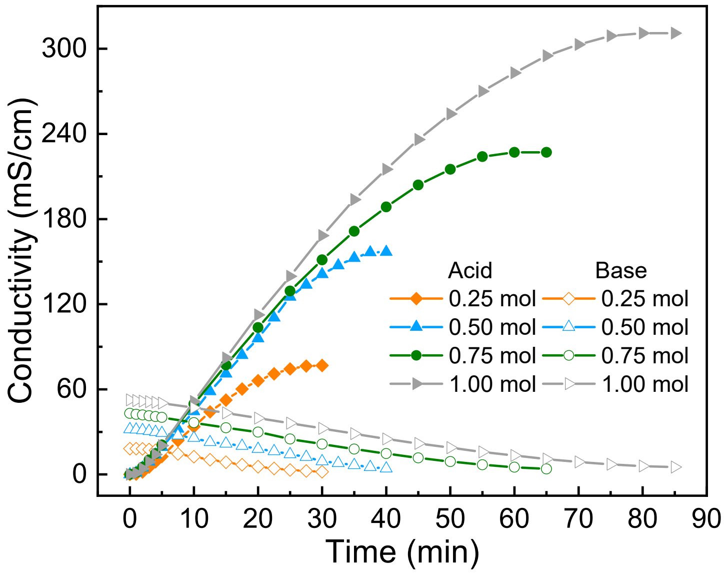

Figure S5. Conductivities of acid and base solutions as a function of time for various feed concentrations.

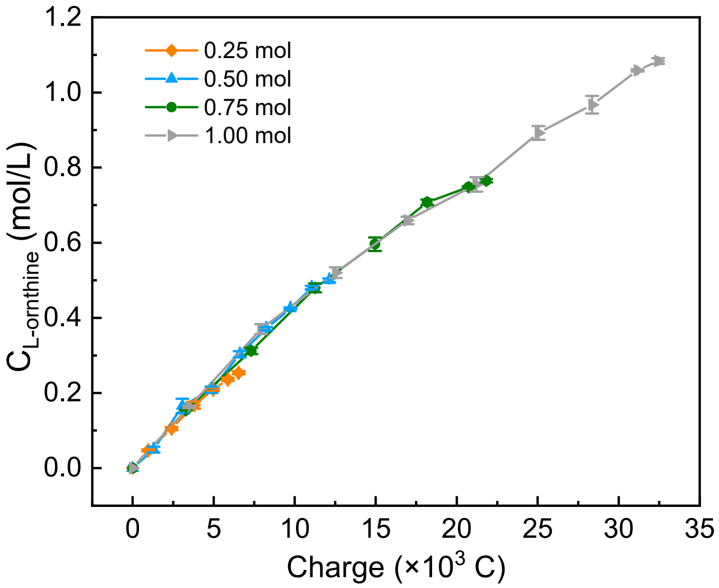

Figure S6. The concentration of L-ornithine in the base solution ($C_{L-ornithine}$) as a function of charge for various feed concentrations.

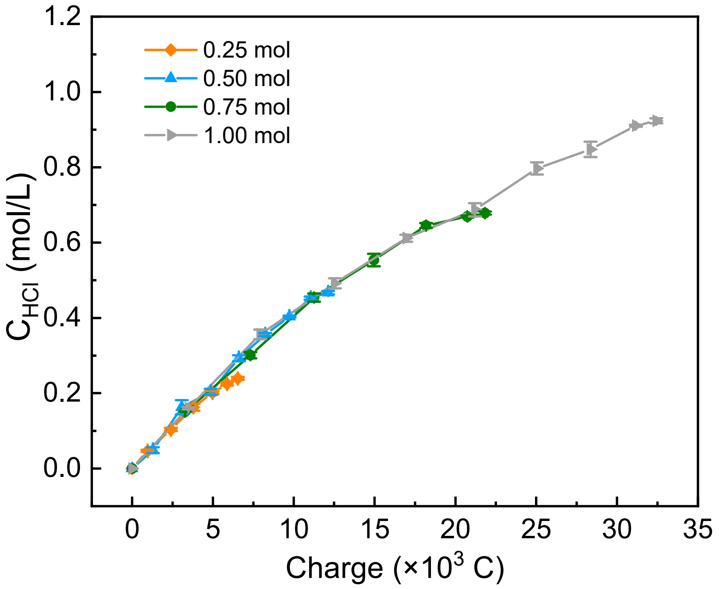

Figure S7. The concentration of HCl in the acid compartment (C_{HCl}) as a function of charge for various feed concentrations.
